# Cultural and Social Determinants of Physical Therapy Rehabilitation in Saudi Arabia: A Narrative Review

**DOI:** 10.3390/healthcare13212773

**Published:** 2025-10-31

**Authors:** Asma Alonazi

**Affiliations:** Department of Physical Therapy and Health Rehabilitation, College of Applied Medical Sciences, Majmaah University, Al-Majmaah 11952, Saudi Arabia; a.alonazi@mu.edu.sa

**Keywords:** culture, adaptation, rehabilitation, physical therapy, Saudi Arabia, cultural competency, vision 2030

## Abstract

**Background:** Modern rehabilitation approaches, encompassing physical, emotional, and social aspects, are gaining momentum in healthcare systems worldwide; however, their acceptance and effectiveness vary across different cultural contexts. **Objective:** This narrative review aims to produce a culturally informed overview of barriers and enablers, highlighting possible strategies to better align evidence-based rehabilitation with Saudi sociocultural realities. **Methods:** Drawing on literature from 2010 to 2024, this narrative review was conducted by searching the peer-reviewed literature from PubMed, Scopus, and the Saudi Digital Library using focused keywords. PICO framework was used to define inclusion and exclusion criteria. Relevant studies addressing cultural influences on rehabilitation adoption were included. **Results:** 1565 articles were initially identified from PubMed, Scopus, and the Saudi Digital Library. After careful screening, eight articles were included in the narrative review. We witnessed key factors relevant to the context of Saudi Arabia deriving health-seeking behaviors to be modesty, fatalism, family support, and religion. Factors possibly associated with the influence of physical therapy rehabilitation were gender, communication barriers, traditional healing practices, and culture and parental involvement. **Conclusions:** In Saudi Arabia, rehabilitation service utilization and practices may be prone to cultural factors. It is of utmost importance that healthcare providers step in and make sure that they sensitize themselves with cultural-specific awareness, knowledge, and competency to deliver optimal rehabilitation healthcare services that meet the standards and needs of the Saudi community.

## 1. Introduction

Chronic noncommunicable diseases account for substantial morbidity and mortality in Saudi Arabia [1,2]. These conditions often result in long-term impairments in physical, social, emotional, and occupational health [3]. Given the limited curative options for diseases such as diabetes, stroke, and brain and spinal cord injury, rehabilitation plays a critical role in restoring function and improving quality of life [4,5].

The persistently high disability in Saudi Arabia is driven by population aging, demographic expansion, and advances in medical technology that increase survival rate [6]. Consequently, the demand for healthcare and rehabilitation services continues to rise, aiming to prevent the progression of chronic diseases to permanent disability or death [7,8]. Numerous factors, including age, gender, body mass index, mental health status, lifestyle habits, educational attainment, environmental conditions, social support, and underlying health status, can significantly influence a patient’s quality of life and well-being [9].

Comprehensive rehabilitation services, including cardiac, orthopedic, occupational, pulmonary, critical care, hearing and speech, substance use, vocational, and perioperative rehabilitation, often integrate physical exercise, dietary management, and behavioral interventions. These approaches have been shown to alleviate symptoms, optimize function, enhance health-related quality of life, and reduce healthcare utilization [10,11].

In the year 2016, Saudi Arabia introduced Kingdom’s ‘Vision 2030’ strategy which also included the Health Sector Transformation Program. The idea of the program is to increase the accessibility to healthcare services by offering universal health coverage, encouraging geographic health equity, and emphasizing electronic medical record utilization and e-health. At the core of this countrywide initiative is innovation targeted at improving the efficiency and quality of patient care services, which also includes rehabilitation services [12].

One critical yet underexplored determinant of rehabilitation service delivery in Saudi Arabia is culture. Cultural and religious beliefs strongly influence healthcare decisions in Saudi Arabia. For example, a study has reported that family-centered decision-making often delays patient autonomy in pursuing healthcare [13]. Gender norms, such as requirement for a male relative’s permission to seek healthcare or travel to a healthcare facility, and the prioritizing of family duties over personal healthcare, have been documented [13]. Religious belief is another key factor. There is this belief that disease from God is an eternal test and, therefore, God should be trusted in managing that disease. This often results in delay in seeking medical support and can also promote the utilization of alternative modes of disease healing like herbal medicine [14].

Advances in rehabilitation, such as robotics, telehealth, virtual reality, and wearable technologies, are gaining traction in physical therapy globally [15]. However, in Saudi Arabia, the adoption of these innovations is not consistent. With personal experience, this inconsistency may not be solely due to infrastructure or cost constraints but could also be due to a misalignment between technological interventions and sociocultural norms. Despite these developments, there is limited synthesis of how cultural and social factors influence the adoption of rehabilitation services in Saudi Arabia. This narrative review aims to produce a culturally informed overview of barriers and enablers, highlighting possible strategies to better align evidence-based rehabilitation with Saudi sociocultural realities.

## 2. Materials and Methods

### 2.1. Study Design

This review was conducted as a narrative review. A narrative approach was deliberately chosen given the limited and heterogeneous body of evidence available on cultural influences in Saudi rehabilitation.

### 2.2. Literature Search and Synthesis

Peer-reviewed articles published between 2010 and 2024 were identified through PubMed, Scopus, and the Saudi Digital Library. As this is a narrative review, the timeframe of 2010–2024 was deliberately chosen to provide a comprehensive overview of the literature from the past decade and beyond. This period was selected because it coincides with major developments in Saudi Arabia’s healthcare system, policy shifts under Vision 2030, and evolving attitudes toward rehabilitation adoption. The following keywords were employed: “rehabilitation”, “physical therapy”, “occupational therapy”, “Saudi culture”, “culture”, “religion”, “health beliefs”, “social”, and “health-seeking behavior”. A simple screening method was used. Titles and abstracts of all retrieved articles were first reviewed for their relevance to rehabilitation and cultural influences in Saudi Arabia. Full texts of potentially relevant studies were then read, and only peer-reviewed articles were included.

### 2.3. Inclusion and Exclusion Criteria

Studies were selected according to predefined PICO inclusion and exclusion criteria (Table 1) to ensure the focus remained on culturally relevant, peer-reviewed evidence from Saudi Arabia and comparable contexts. The Population (P) of interest included adults in Saudi Arabia, and where relevant, the wider Gulf/Arab region, who were engaged in or eligible for physical therapy and rehabilitation services. The Intervention (I) of interest was the adoption and utilization of modern rehabilitation and physical therapy practices. The Comparison (C) was not a formal control group but rather traditional cultural norms, beliefs, and practices that may influence rehabilitation adoption (e.g., gender roles). The Outcomes (O) of interest included reported barriers and enablers to rehabilitation adoption, with emphasis on how sociocultural realities shaped access, engagement, and acceptance of services. No restrictions were placed on the type of articles included due to the limited number of available studies.

## 3. Results

**Figure 1:**
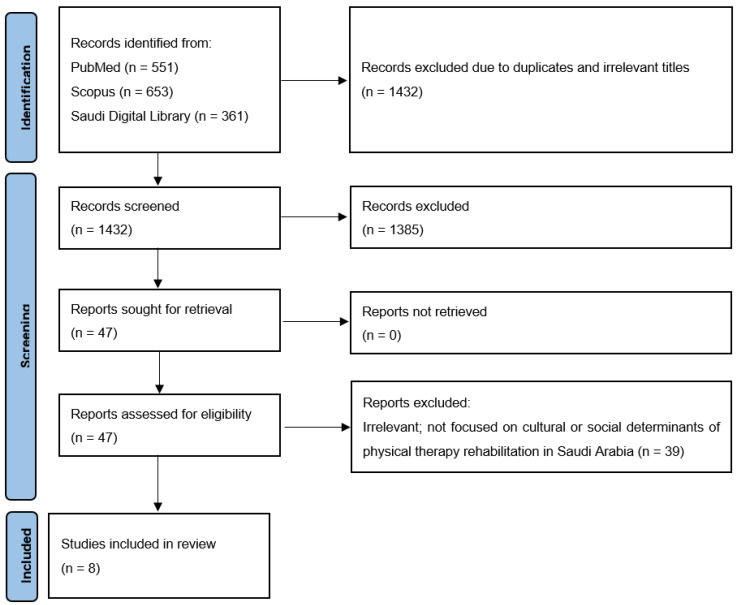
Study screening and selection.

Overall, 1565 articles were initially identified. After removing duplicates and irrelevant articles, 1518 articles were excluded during the initial screening of titles and abstracts, resulting in 47 articles remaining for full-text review. Ultimately, eight articles [14,16,17,18,19,20,21,22] were deemed suitable for inclusion in the narrative review. Figure 1 illustrates the flowchart detailing the search results and the process of article selection.

### 3.1. Healthcare Practices in Saudi Arabia—Cultural and Social Context

Table 2 summarizes studies examining the cultural and social context in healthcare practices in Saudi Arabia.

#### 3.1.1. Modesty

Modesty is among the most deeply rooted cultural values and beliefs in Saudi Arabia. It generally required both men and women to dress in a manner that is non-revealing and represents decency. It is important to understand that this cultural value has substantial impact on how women practice healthcare in Saudi Arabia. With traditional approaches in Saudi Arabia, women are often supposed to be attended to by a male family member or a custodian for their clinic or hospital visits. This cultural custom greatly reflects the value system of Saudi Arabia that women must be secured from the stare of strangers and men in the family are considered their caretakers. This cultural custom can present a significant challenge for women in healthcare settings [16].

#### 3.1.2. Fatalism

Yet another important healthcare belief in Saudi Arabia is the notion of fatalism. In Saudi Arabia, it is believed that health and disease are fated by God (Allah) and, therefore, going to the hospital to receive treatment is akin to going against the wish of God. This can substantially prevent people from attending to healthcare matters timely which can eventually lead to unnecessary delay in diagnosis and management of the disease. Interestingly, high mean fatalism scores have been observed among Saudi health sciences students, which also reflects the role of cultural influence [17].

#### 3.1.3. Family Support

Family support is fairly strong in Saudi Arabia. Therefore, during the period of illness, it is very common in Saudi Arabian culture that family members come forward to provide care and support to the sick. This may also discourage the patient from visiting the hospital for appropriate healthcare. There is no doubt that emotional support is essential while the patient is sick; however, to manage the disease, medical treatment is equally critical. Not receiving appropriate treatment will only lead to unnecessary delay and worsen the prognosis for the patients. For example, one study from Saudi Arabia reported significant antibiotic self-medication (54%) instead of consulting a clinician [18].

#### 3.1.4. Religion

Another significant influence in the lives of Saudi Arabian people is religion and religious practice which can also impact health-related behaviors and attitudes. The idea of purity and cleanliness and how it impacts health is an important religious belief amongst Saudi Arabian people. In the faith of Islam, purity and cleanliness is regarded as half of the faith and, therefore, the followers of Islam (Muslims) are supposed to adequately follow and maintain hygiene practices for both the person and their surroundings. This belief is positively associated with routine healthcare practices because it motivates people to exercise caution and thereby prevent disease [14].

### 3.2. Cultural Influence on Physical Therapy Rehabilitation

Table 3 summarizes studies examining possible cultural influence in physical therapy rehabilitation in Saudi Arabia.

#### 3.2.1. Gender-Specific Challenges

In Saudi Arabia, managing disease rehabilitation for women may present unique challenges due to cultural norms and societal expectations. Women are often expected to prioritize family responsibilities, such as caregiving and household duties, which can interfere with attending physical therapy sessions. In addition, women are not allowed to consult male physiotherapists or any physiotherapist without the supervision of a husband or a male family member. This also denies them the right of making treatment decisions on their own. These concerns have been raised by female physiotherapists in a research study by Altamimi 2015 [21].

#### 3.2.2. Communication Barriers

Communication barriers with physiotherapy professionals can also hinder progress, making it difficult for patients to express their concerns and needs. For example, according to patients’ perspective, one study found factors possibly associated with communication barriers in physical/occupational therapist patients such as limited consultancy time, fewer consultancies, and the therapist’s gender [20].

#### 3.2.3. Reliance on Traditional Healing Practices

Patients may prefer traditional healing methods, such as herbal medicine, skin cauterization, or spiritual healing. A research study by Mohammad et al. (2015) reported a 67% prevalence of use of traditional medicine among Saudi patients with diverse neurological conditions [22]. The traditional medicine methods included cupping, herbs, and skin cauterization.

#### 3.2.4. Culture and Parental Involvement

When it comes to pediatric patients with physical disabilities or impairments, the role of parents in the process of rehabilitation becomes indispensable. Family-centered care is highly regarded as an ideal approach in pediatric rehabilitation. A study from Saudi Arabia by Alharbi and Albalwi (2023) assessed the factors that can possibly influence the perception of parents for family-centered care for children with cerebral palsy [19]. Authors found the child’s gender, education level of the mother, and residence region to be the influential factors. Earlier research studies did not report the child’s gender to be a factor that can affect the perception of parents towards family-centered care. One plausible explanation for this could be cultural diversity. In Saudi Arabia and other Gulf Cooperation Council countries, parents are quite sensitive and overprotective of the needs of their female child [19].

## 4. Discussion

Undoubtedly, religion and culture are an integral and important part of how a society practices healthcare. This significantly impacts how one individual perceives his or her health and behavior and attitude towards health and, in fact, the healthcare system. The Kingdom of Saudi Arabia is no different. In Saudi Arabia, religion and culture have immense impact on health-related practices. Among many Islamic countries around the world, Saudi Arabia is one of the most conservative Islamic countries. The country has a distinctive religious and cultural practice that significantly influences health-related behaviors of its citizens. Most of the cultural norms and customs in Saudi Arabia emanate from the religion of Islam and reflect teachings of the Quran (the Holy Book) [23]. In the present narrative review study, we witnessed key factors relevant to the context of Saudi Arabia deriving health-seeking behaviors to be modesty [16], fatalism [17], family support [18], and religion [14]. On the other hand, factors possibly associated with influence of physical therapy rehabilitation were gender [21], communication barriers [20], traditional healing practices [22], and culture and parental involvement [19].

To address such challenges in Saudi Arabia and effectively enhance utilization of rehabilitation services and patients outcomes, inspiration can be drawn from a review study on cultural adaptation in cardiac rehabilitation [24,25]. A recent review by Marchand et al. (2024) summarized the scientific evidence on the cultural adaptation of cardiac rehabilitation programs for indigenous patients, its success in some nations, and key strategies to be implemented in Canada [25]. One of the key components of care of patients with cardiovascular disease is cardiac rehabilitation, which has been shown to significantly diminish clinical complications and associated mortality, enhance health-related quality of life, and reduce healthcare cost [26,27]. Canada has a rich colonial history and, therefore, indigenous people dwelling in Canada have a higher prevalence of cardiovascular diseases and cardiovascular-specific deaths. Intriguingly, the indigenous community in Canada possess a distinctive geographical, sociocultural, and historical background that prevents their accessibility to ideal cardiovascular care, which also includes cardiac rehabilitation. This disparity significantly affects the clinical outcomes for such populations. For instance, studies have confirmed that indigenous patients may hesitate to visit family physicians, cardiologists, or internists post-myocardial infarction or heart failure complication, possibly indicating exposure to healthcare racism and associated transgenerational psychological trauma that might have led to lack of trust in the healthcare system [28,29]. Culturally tailored cardiac rehabilitation programs have demonstrated success in Australia, Canada, and New Zealand, showing acceptance among indigenous patients and improved outcomes. According to the authors, strategies that could prove to be successful in the implementation of culturally adapted cardiac rehabilitation programs are as follows: (1) Engagement of individuals belonging to indigenous communities for cardiac rehabilitation program development and leadership. (2) Preparation of healthcare professionals to be culturally sensitive in their cardiac rehabilitation practices. (3) Encouragement healthcare professionals to implement and promote conventional practices, such as maintaining a traditionally healthy diet and recognizing land-based physical activity as forms of exercise, has been shown to be effective in delivering successful cardiac rehabilitation among indigenous community.

### 4.1. Possible Solutions to Culture and Rehabilitation Practice Challenge in Saudi Arabia

Healthcare professionals are often encouraged to acquire and implement cultural competency while caring for patients. However, offering a culturally competent rehabilitation service can still be a mammoth task due to a handful of the following reasons: (1) patient’s culture can significantly affect their values and belief systems and behaviors towards health [30,31], (2) rehabilitation interventions are usually designed to suit the cultural value and belief systems of the major population which could consequently disregard the minority population with diverse cultural backgrounds [32,33,34], and (3) issues of assessment bias can arise which could result in misdiagnosis among minority cultural backgrounds due to the incorrect interpretation of competence of patients [35].

To improve the acceptance of rehabilitation practices among patients in Saudi Arabia, the outlined approaches can be implemented [35,36,37,38]: (1) Healthcare professionals associated with rehabilitation services must receive specialized training to be culturally competent to understand and respect Saudi cultural value and belief systems. (2) Practitioners in rehabilitation services, such as physical therapists, must give consideration to their own multifaceted value and belief systems and prejudgments that may influence the care of the patient. For this, practitioners should take regular feedback from patients and learn patients’ perspective to make appropriate changes in their attitudes and practice to be culturally efficient in care provision. (3) It is equally important to acknowledge the issue, set targets, and design customized rehabilitation programs to cater to diverse cultural backgrounds. (4) Gender-specific rehabilitation programs can be established to respect cultural customs and norms while delivering efficient patient care. (5) Patients can be actively involved in goal setting to improve their satisfaction, motivation, and physiological outcomes while lessening restrictions in an individual’s functioning. (6) Practitioners must be encouraged to undertake a social and cultural examination as well as to practice and provide culturally competent rehabilitation services. To obtain insight of the patient’s culture, their historical backdrop, role of religious practices, and conventional healing techniques, informing the role of cultural background to the patient, making strong therapeutic associations with patient, cross-cultural communication with patient, and asking patients to embrace their value and belief system will assist rehabilitation practitioners in delivering culturally focused care and practice. (7) Another way to foster a culturally efficient rehabilitation work ecosystem and a diverse cadre of professionals is to prepare practitioners to offer services to minority groups, design biodata forms that gather information relevant to different languages and cultural and ethnic backgrounds, including that of family members, connect patients and practitioners based on identical cultural backgrounds, and integrate the concept of culturally competent rehabilitation into graduate-level degree programs. (8) To increase the acceptance and compliance among patients, conventional healing techniques can be combined with modern rehabilitation techniques. (9) Finally, community leaders and religious figures can also be engaged to promote rehabilitation services and can assist in bridging the cultural gap.

### 4.2. Future Recommendations and Research Directions

It is imperative that culturally sensitive care provision must be actively integrated into the modern physical therapy and rehabilitation practices in Saudi Arabia. The following recommendations can be outlined to emphasize effective and culturally focused rehabilitation interventions: (1) Upcoming research must focus on significance of cultural competence in rehabilitation practices by designing physical therapy interventions that coalesce with distinct sociocultural norms in Saudi Arabia. (2) Linguistic barriers, in the context of non-Arabic speaking individuals, must be addressed and conventional healing practices that reflect Saudi customs and beliefs must be incorporated to improve the outcomes associated with rehabilitation programs. (3) Socioeconomic disproportions impact accessibility to healthcare and, therefore, should also be dissected to guarantee fairness in rehabilitation services across various parts and groups in Saudi Arabia. (4) Inclusive rehabilitation practices should be designed and investigated, keeping in mind the gender-oriented cultural setting of Saudi Arabia. (5) Saudi society is heavily based on families and communities and hence, they should be engaged to offer holistic support and improve patient outcomes. (6) Furthermore, culturally sensitive assessment tools must be established and validated. (7) Likewise, clinicians must be equipped to utilize and provide culturally sensitive rehabilitation approach and care, respectively. (8) Future research should focus on longitudinal studies to assess the long-term impact of culturally adapted rehabilitation practices. (9) The role of technology in enhancing culturally sensitive care and investigating the perspectives of patients and healthcare providers should be a priority as they can provide deeper insights into effective cultural adaptation strategies. (10) Finally, it is equally important to impart and sensitize entry-level physical therapy program students and explore the concept of culturally adapted rehabilitation practices.

### 4.3. Limitation and Strength

This review has several inherent limitations. Firstly, it is a narrative review and, therefore, lacks a systematic search and appraisal process, which may have introduced selection bias and limit reproducibility. Secondly, there is a lack of original studies from Saudi Arabia, which confines the depth of analysis, and a reliance on descriptive reports or other review papers which might affect the strength of evidence. Finally, due to the narrative nature of the review and lack of original research studies, the findings must be interpreted as contextual insights rather than definitive conclusions. Nevertheless, the review is strengthened by the fact that it attempted to synthesize scattered and limited evidence into a single, accessible overview, providing a culturally informed lens on rehabilitation in Saudi Arabia.

## 5. Conclusions

Cultural beliefs strongly influence how people in Saudi Arabia view health, illness, and treatment. These beliefs may also affect how rehabilitation services, such as physical therapy, are used. Therefore, healthcare providers must understand and respect local cultural values when offering rehabilitation care. Developing cultural awareness and competence helps providers deliver more effective and acceptable services. By addressing cultural barriers, they can improve patient outcomes and increase the use of modern rehabilitation services in Saudi Arabia.

## Figures and Tables

**Table 1:** PICO Framework for Study Selection.

PICO	Criteria
Population (P)	Adults in Saudi Arabia, and where relevant, the wider Gulf/Arab region, who were engaged in or eligible for physical therapy and rehabilitation services.
Intervention (I)	Adoption and utilization of modern rehabilitation and physical therapy practices.
Comparison (C)	Not a formal comparator group; instead, traditional cultural norms, beliefs, and practices (e.g., gender roles) that may influence rehabilitation adoption.
Outcome (O)	Reported barriers and enablers to rehabilitation adoption, with particular focus on the impact of Saudi sociocultural realities on access, engagement, and acceptance of services.

**Table 2:** Studies correlating cultural and social context in healthcare practices in Saudi Arabia.

Study	Type of Study	Variable	Relevant Findings
[16]	Policy Paper	Modesty	Gender norms in Saudi Arabia focus on woman’s modesty and the protection of their sexuality.
[17]	Cross-sectional	Fatalism	High mean fatalism scores among Muslim health sciences students from Saudi Arabia.
[18]	Cross-sectional	Family Support	Antibiotic self-medication prevalence of 54% in children by parents.
[14]	Qualitative	Religion	Islam emphasizes preserving good health and seeking a cure for any sickness.

**Table 3:** Studies concerning cultural influence in physical therapy rehabilitation.

Study	Type of Study	Variable	Results
[21]	Qualitative	Gender-Specific Challenges	Lack of rights for women to make their treatment decisions according to physiotherapists.
[20]	Cross-sectional	Communication Barriers	Among several factors, gender was also found to be the communication barrier in physical/occupational therapists according to patients.
[22]	Cross-sectional	Traditional Healing Practices	67% prevalence of use of traditional medicine among Saudi patients with diverse neurological conditions.
[19]	Cross-sectional	Culture and Parental Involvement	Child’s gender is a factor that can influence the perception of parents for family-centered care for children with cerebral palsy.

## Data Availability

No new data were created or analyzed in this study. Data sharing is not applicable to this article.
